# Influenza H3 hemagglutinin vaccine with scrambled immunodominant epitopes elicits antibodies directed toward immunosubdominant head epitopes

**DOI:** 10.1128/mbio.00622-23

**Published:** 2023-07-19

**Authors:** Shiho Chiba, Huihui Kong, Gabriele Neumann, Yoshihiro Kawaoka

**Affiliations:** 1 Influenza Research Institute, Department of Pathobiological Sciences, School of Veterinary Medicine, University of Wisconsin-Madison, Madison, Wisconsin, USA; 2 Division of Virology, Department of Microbiology and Immunology, Institute of Medical Science, University of Tokyo, Tokyo, Japan; 3 The Research Center for Global Viral Diseases, National Center for Global Health and Medicine Research Institute, Tokyo, Japan; 4 Pandemic Preparedness, Infection and Advanced Research Center (UTOPIA), The University of Tokyo, Tokyo, Japan; Johns Hopkins University Bloomberg School of Public Health, Baltimore, Maryland, USA; Johns Hopkins University Bloomberg School of Public Health, Baltimore, Maryland, USA

**Keywords:** influenza A (H3N2) viruses, universal vaccines, ferret, immunosubdominant epitopes

## Abstract

Vaccination is the most effective countermeasure to reduce the severity of influenza. Current seasonal influenza vaccines mainly elicit humoral immunity targeting hemagglutinin (HA). In particular, the amino acid residues around the receptor-binding site in the HA head domain are predominantly targeted by humoral immunity as “immunodominant” epitopes. However, mutations readily accumulate in the head domain due to high plasticity, resulting in antigenic drift and vaccine mismatch, particularly with influenza A (H3N2) viruses. A vaccine strategy that targets more conserved immunosubdominant epitopes is required to attain a universal vaccine. Here, we designed an H3 HA vaccine antigen with various amino acids at immunodominant epitopes of the HA head domain, termed scrambled HA (scrHA). In ferrets, scrHA vaccination induced lower serum neutralizing antibody levels against homologous virus compared with wild-type (WT) HA vaccination; however, similar levels of moderately neutralizing titers against antigenically distinct H3N2 viruses were observed. Ferrets vaccinated with scrHA twice and then challenged with homologous or heterologous virus showed the same level of reduced virus shedding in nasal swabs as WT HA-vaccinated animals but reduced body temperature increase, whereas WT HA-vaccinated ferrets exhibited body temperature increases similar to those of mock-vaccinated animals. scrHA elicited antibodies against HA immunodominant and -subdominant epitopes at lower and higher levels, respectively, than WT HA vaccination, whereas antistalk antibodies were induced at the same level for both groups, suggesting scrHA-induced redirection from immunodominant to immunosubdominant head epitopes. scrHA vaccination thus induced broader coverage than WT HA vaccination by diluting out the immunodominancy of HA head epitopes.

**IMPORTANCE:**

Current influenza vaccines mainly elicit antibodies that target the immunodominant head domain, where strain-specific mutations rapidly accumulate, resulting in frequent antigenic drift and vaccine mismatch. Targeting conserved immunosubdominant epitopes is essential to attain a universal vaccine. Our findings with the scrHA developed in this study suggest that designing vaccine antigens that “dilute out” the immunodominancy of the head epitopes may be an effective strategy to induce conserved immunosubdominant epitope-based immune responses.

## INTRODUCTION

Influenza viruses have caused annual epidemics and pandemics every few decades in the human population, leading to serious global morbidity and mortality. The most effective countermeasure against virus-caused illness is vaccination. The current seasonal influenza vaccines mainly elicit humoral immune responses against hemagglutinin (HA) protein, a surface glycoprotein of the virus. The amino acid (AA) residues around the receptor-binding site (RBS) in the HA head domain are predominantly targeted by humoral immunity as “immunodominant” epitopes, whereas the membrane-proximal stalk domain is immunosubdominant. Due to the high plasticity of the HA head domain, however, mutations readily accumulate in this domain, resulting in the frequent antigenic drift of the virus. Therefore, influenza vaccine virus strains must be correspondingly revised and updated. Vaccines that target more conserved regions, including the stalk domain and relatively conserved immunosubdominant epitopes in the head domain, are urgently needed to elicit broadly cross-reactive immune responses across strains (i.e., universal influenza vaccines).

To target the more conserved immunosubdominant epitopes in HA, several different strategies of vaccine antigen design have been attempted based on influenza A H1 HA, H3 HA, or influenza B HA, such as chimeric HA (cHA) or “mosaic” HA composed of the immunodominant head domain or amino acid residues derived from exotic HA subtypes (1 - 6), hyper-glycosylated HAs to shield immunodominant epitopes (7, 8), and headless HAs (9 - 16). Here, based on H3 HA, we devised another strategy to design a universal vaccine candidate with the idea of “diluting out” the immunodominancy of the head domain: scrambled HA (scrHA), which has a variety of amino acids at the antigenically dominant loci in the HA head domain. The immune responses elicited by scrHA were examined in ferrets.

## RESULTS

### Isolation of Tokyo/14 17-AA mutant HAs with antigenic diversity at the immunodominant head as vaccine antigen candidates

**FIG 1:**
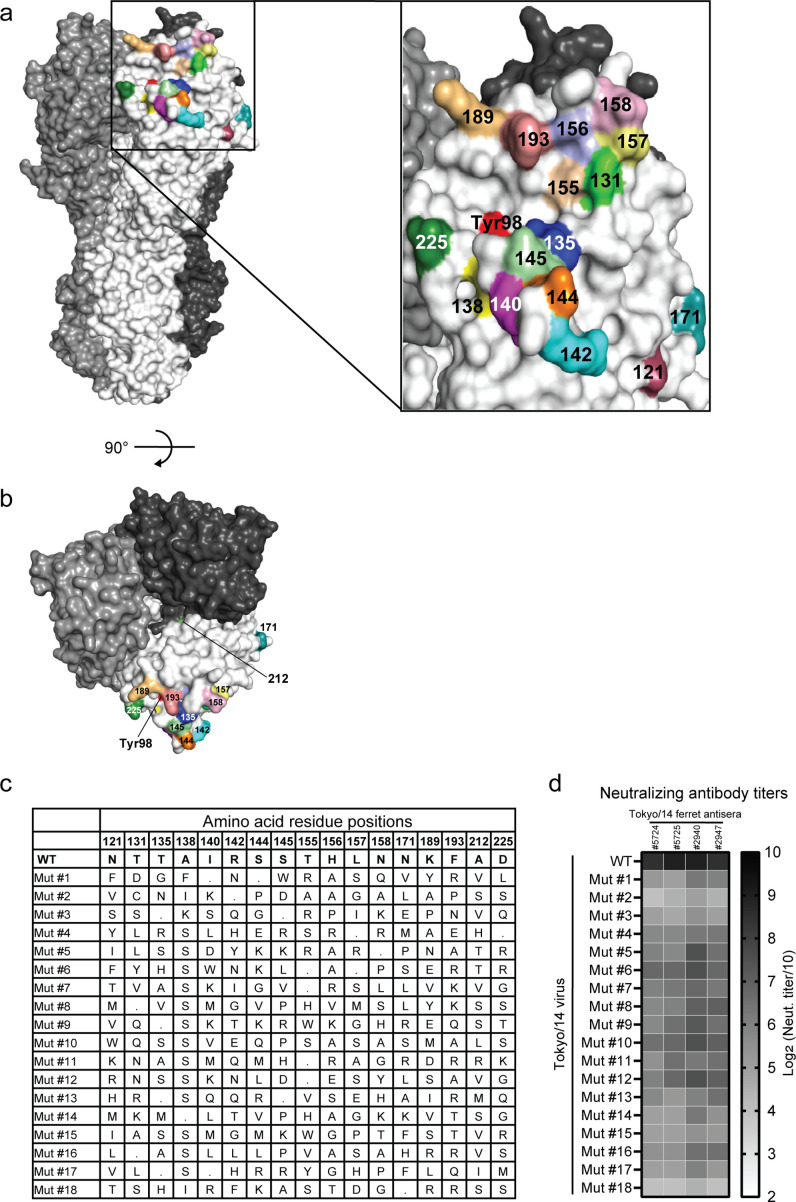
Preparation of Tokyo/14 17-AA scrambled HA vaccine. Side view (**a**) and top view (**b**) of the 17-AA mutagenesis positions. The 17 amino acid positions selected for mutagenesis are mapped in different colors on an HA monomer (shown in white; the other two monomers are shown in gray and black) of the A/Victoria/361/2011 (H3N2) influenza virus HA structure (PDBID: 4O5N). The amino acid residue at the receptor-binding pocket (Tyr98) is shown in red. (**c**) The table shows the amino acid residues at the 17 mutagenesis positions in wild-type A/Tokyo/UT-IMS2-1/2014 (Tokyo/14) HA and the amino acid substitutions in mutant HA #1–18. Dots indicate the identical amino acid residues to that found in wild-type HA. (**d**) Neutralizing antibody titers against Tokyo/14 WT and Mut #1 to #18 viruses were examined with A/Tokyo/UT-IMS2-1/2014 ferret antisera from four individual animals (ferret IDs 5724, 5725, 2940, and 2947).

The antigenic changes in H3 HA since the emergence of H3N2 viruses have been mainly caused by amino acid substitutions at a limited number of antigenically dominant locations in the RBS of the HA (17). We compiled the amino acid locations that have been shown to affect the antigenic properties of group 1 HAs (H1 and H5) (18 - 20) as well as H3 HA (17) and selected 17 AA positions (121, 131, 135, 138, 140, 142, 144, 145, 155, 156, 157, 158, 171, 189, 193, 212, and 225; H3 numbering) in the HA head domain, which have been reported to critically affect the antigenicity of the protein (Fig. 1a and b). We previously established a method to introduce random amino acid substitutions at selected positions in the HA head domain to obtain a library of viruses expressing genetically divergent HAs (19, 20). By utilizing this strategy, we generated A/Tokyo/UT-IMS2-1/2014 (Tokyo/14; H3N2, clade 3 c.2a) 17-AA mutant library virus. Briefly, a synthesized DNA fragment encoding Tokyo/14 HA with codon “NNK” (N: A, T, G, or C; K: G, or T) at the selected 17 codons and wild-type (WT) codons in the remaining portion was cloned into a plasmid vector under the control of the RNA polymerase I promoter and terminator to obtain the HA library plasmid. Then, the HA library plasmid was rescued with the homologous Tokyo/14 NA and remaining viral RNA segments for the internal genes from high-yield A/Puerto Rico/8/34 (PR8-HY) (21) to generate the Tokyo/14 HA library virus. From this library virus, antigenically escaped mutant viruses were isolated by either plaque assays supplemented with mixed human convalescent sera (collected in 2015–2016) or serial passaging of the library viruses followed by cloning and antigenic analyses of HAs (see details in Supplementary Note 1). In total, 18 different clones of Tokyo/14 17-AA mutant viruses (Mut #1 to #18) that had individually various amino acid substitutions at the 17-AA positions were isolated (Fig. 1c). Notably, all 18 mutant viruses were replication competent in cells, demonstrating that the mutant HAs were functional despite the multiple amino acid substitutions around the RBS. The 18 mutant viruses were analyzed in micro-neutralization assay to examine their reactivity against Tokyo/14 virus ferret antisera. All mutant viruses showed 2.8- to 56-fold lower reactivity against the Tokyo/14 ferret antisera compared to that of the wild-type Tokyo/14 virus (Fig. 1d), indicating that they are antigenically distinct from the wild-type Tokyo/14 HA.

### Scrambled HA (scrHA) vaccination elicits equivalent neutralizing antibody titers against homologous and heterologous viruses in ferrets

**FIG 2:**
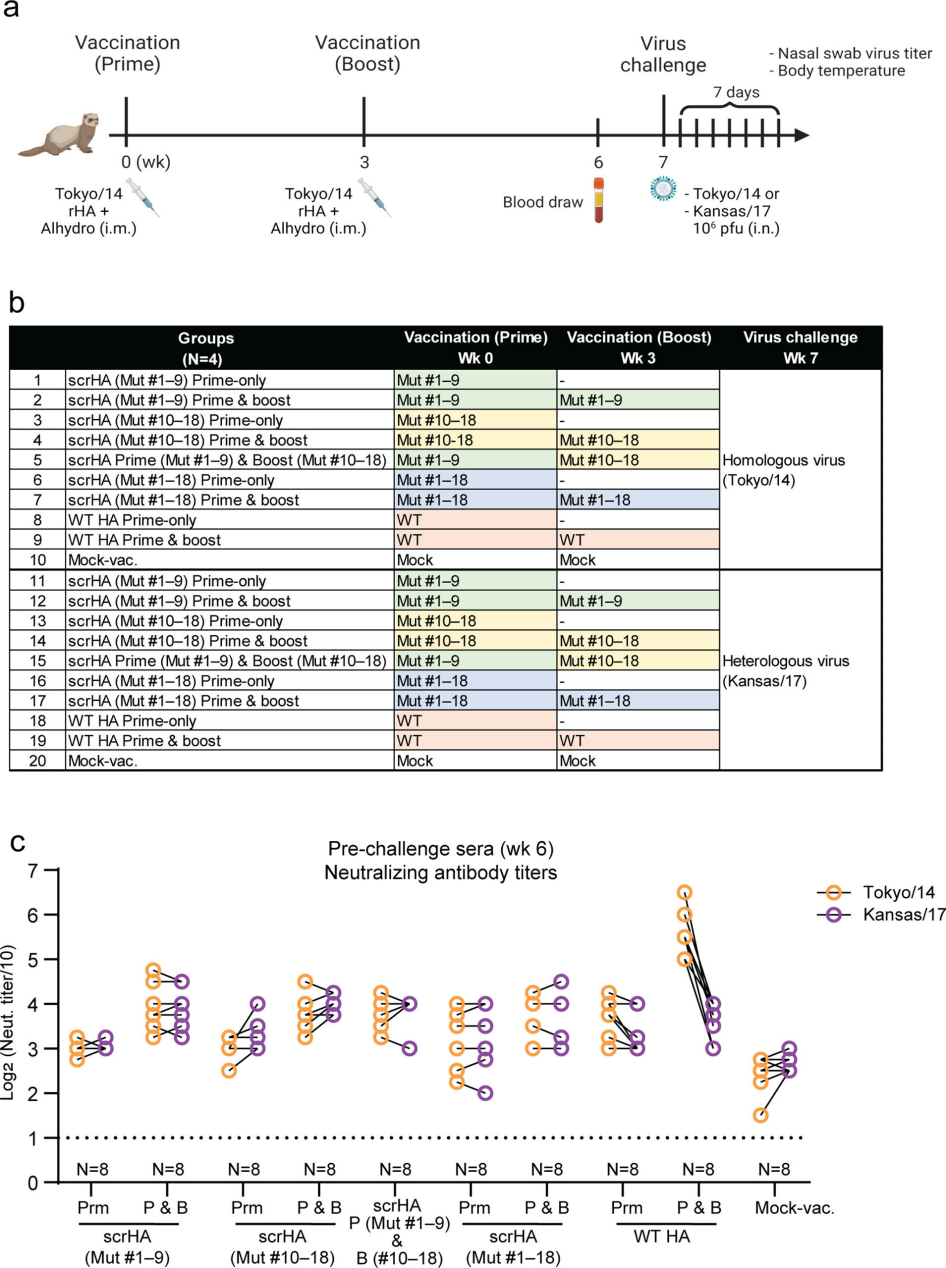
Experimental design for the scrHA ferret vaccination study and pre-challenge serum neutralizing antibody titers. (**a**) Timeline and (**b**) vaccination regimens in the ferret virus challenge study. Groups of ferrets (*N* = 4/group) were intramuscularly (i.m.) vaccinated with Tokyo/14 recombinant HA (rHA) adjuvanted with Alhydrogel as part of a prime-only or prime-and-boost regimen. Pre-challenge sera were collected at 6 wk post-prime vaccination. Then, the animals were intranasally (i.n.) challenged with 10^6^ plaque-forming units (pfu) of homologous A/Tokyo/UT-IMS2-1/2014_PR8HY (Tokyo/14) virus or heterologous A/Kansas/14/2017_PR8HY virus (Kansas/17). Mut, mixture of mutants; –, not applicable. (**c**) All ferrets were bled at 6 wk (pre-challenge) and serum neutralizing antibody titers against Tokyo/14 virus and Kansas/17 were analyzed in micro-neutralization assays. Data points show the values of individual animals connected by lines for each animal. Data shown are the geometric means of duplicates. Dashed lines represent the detection limit of the assay. Prm, prime-only regimen; P & B, prime-and boost-regimen. Illustration created with BioRender.com.

Next, we examined the effects of the mutant HAs as vaccine antigens in ferrets. To focus on the effects of HA and eliminate contributions of other viral proteins, recombinant HA protein vaccine was used. The HA genes from the 18 mutant viruses or WT Tokyo/14 virus were individually cloned into expression plasmids and expressed as a soluble form of HA in a cell line. Equal amounts of purified mutant HA proteins were mixed to formulate mutant mixture vaccines, which we termed “scrambled HA (scrHA)” vaccines. Mixtures of nine different mutants [i.e., mutants (Mut) #1–9 or Mut #10–18] or all 18 mutants (Mut #1–18) were formulated. Ferrets were intramuscularly immunized with 15 µg of Tokyo/14 scrHA or WT HA vaccine adjuvanted with Alhydrogel by using either a prime-only regimen or a prime-and-boost regimen (Fig. 2a and b). At 6 wk post-prime, serum titers were analyzed in neutralization assay against the homologous virus Tokyo/14 and the antigenically distinct heterologous virus A/Kansas/14/17 (Kansas/17, clade 3 c.3a1) (Fig. 2c). The animals that received WT HA through the prime-and-boost regimen showed the highest neutralizing titer against Tokyo/14 virus [geometric mean titers (GMT): 320–905], whereas two- to eightfold lower neutralizing titers were observed against the heterologous Kansas/17 virus in the same animals (Fig. 2c). The WT HA prime-only vaccinated group showed similar titers against Tokyo/14 virus and Kansas/17 virus, suggesting that boost vaccination with WT HA effectively elicited strain-specific neutralizing antibodies against the homologous virus. The groups that received scrHA vaccine twice (prime-and-boost with Mut #1–9, Mut #10–18, or Mut #1–18, or Mut #1–9 prime and Mut #10–18 boost) showed GMT of 80–269 against Tokyo/14 virus, and the equivalent titers were also observed against Kansas/17 virus (Fig. 2c). The prime-only regimen of scrHA vaccination induced slightly lower titers than the prime-and-boost regimen against both viruses. These results suggest that scrHA vaccination is not as potent as WT HA in eliciting serum neutralizing antibodies against homologous virus but can induce equivalent levels of neutralizing titers against antigenically distinct virus.

### scrHA vaccination reduces virus replication and disease symptoms in ferrets

**FIG 3:**
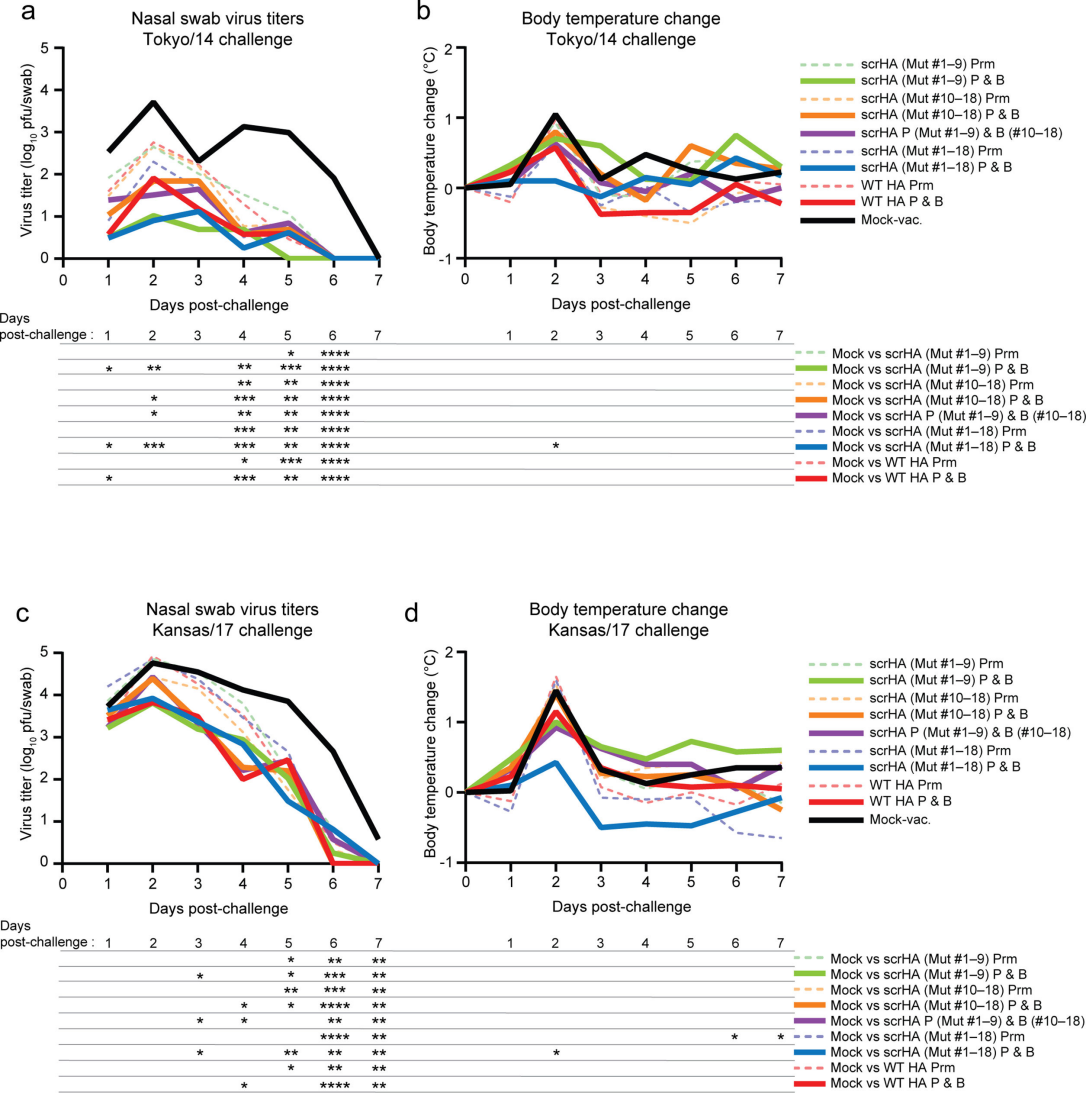
Virus replication and clinical symptoms in ferrets after scrHA vaccination. Vaccinated or mock-vaccinated ferrets (*N* = 4/group) were intranasally infected with 10^6^ pfu of homologous A/Tokyo/UT-IMS2-1/2014_PR8HY (Tokyo/14) virus (**a and b**) or heterologous A/Kansas/14/2017_PR8HY (Kansas/17) virus (**c and d**). Virus titers in nasal swabs (**a and c**) and body temperature increase (**b and d**) as a clinical symptom were monitored for 7 days post-challenge. Data represent the means of each group. Statistical analyses (each vaccinated group was compared to mock-vaccinated group) were performed by using a one-way analysis of variance and corrected for multi-group comparison by using Dunnett’s test. (*P < 0.05; **P < 0.01; ***P < 0.001; ****P < 0.0001). Data of individual animals are shown in Supplementary Figures 1–4. Prm, prime-only regimen; P & B, prime-and-boost regimen.

Vaccinated or mock-vaccinated animals were intranasally challenged with 10^6^ plaque-forming units (pfu) of vaccine-homologous Tokyo/14 (clade 3 c.2a) virus or antigenically distinct heterologous virus Kansas/17 (clade 3 c.3a1). The challenge viruses were generated by reverse genetics with the PR8 high-yield backbone (21) to eliminate the effect of the difference in contributions to virus replication and/or clinical symptoms in ferrets of the genes encoding the viral internal proteins of the homologous and heterologous viruses. Nasal swabs and body temperature were taken daily for 7 days to evaluate virus replication and symptom severity (Fig. 3), since these viruses do not replicate well or cause inflammatory lesions in ferret lungs. Upon challenge with Tokyo/14 virus, the scrHA and WT HA prime-and-boost vaccinated groups showed significantly reduced virus titers in nasal swabs compared to the mock-vaccinated group on 1–2 days post-challenge and eliminated the virus 1–2 days earlier than the mock-vaccinated group (Fig. 3a). Prime-only regimen vaccination with either scrHA or WT HA led to significantly lower nasal swab titers at later timepoints compared with the mock-vaccinated group (day 4–6; Fig. 3a). Upon heterologous Kansas/17 virus challenge, the groups that received scrHA twice showed significantly reduced nasal virus titers compared to the mock-vaccinated group at four timepoints including day 3, whereas the WT HA prime-boosted group showed reduced nasal virus titers compared to the mock-vaccinated group at three timepoints (Fig. 3c). The body temperature of the mock-vaccinated group peaked on day 2 upon either Tokyo/14 or Kansas/17 virus challenge (Fig. 3b and d). Notably, only the scrHA (Mut #1–18) prime-and-boosted group showed significantly lower body temperature compared with the mock-vaccinated group upon either virus challenge on day 2 (Fig. 3b and d). These results demonstrate that a prime-boost vaccination with scrHA, but not with WT HA, effectively prevented an increase in body temperature upon challenge with homologous or heterologous virus, whereas the replication of those viruses was similarly reduced in both vaccine groups.

### Not high but broadly cross-reactive neutralizing antibodies can be induced by scrHA

**FIG 4:**
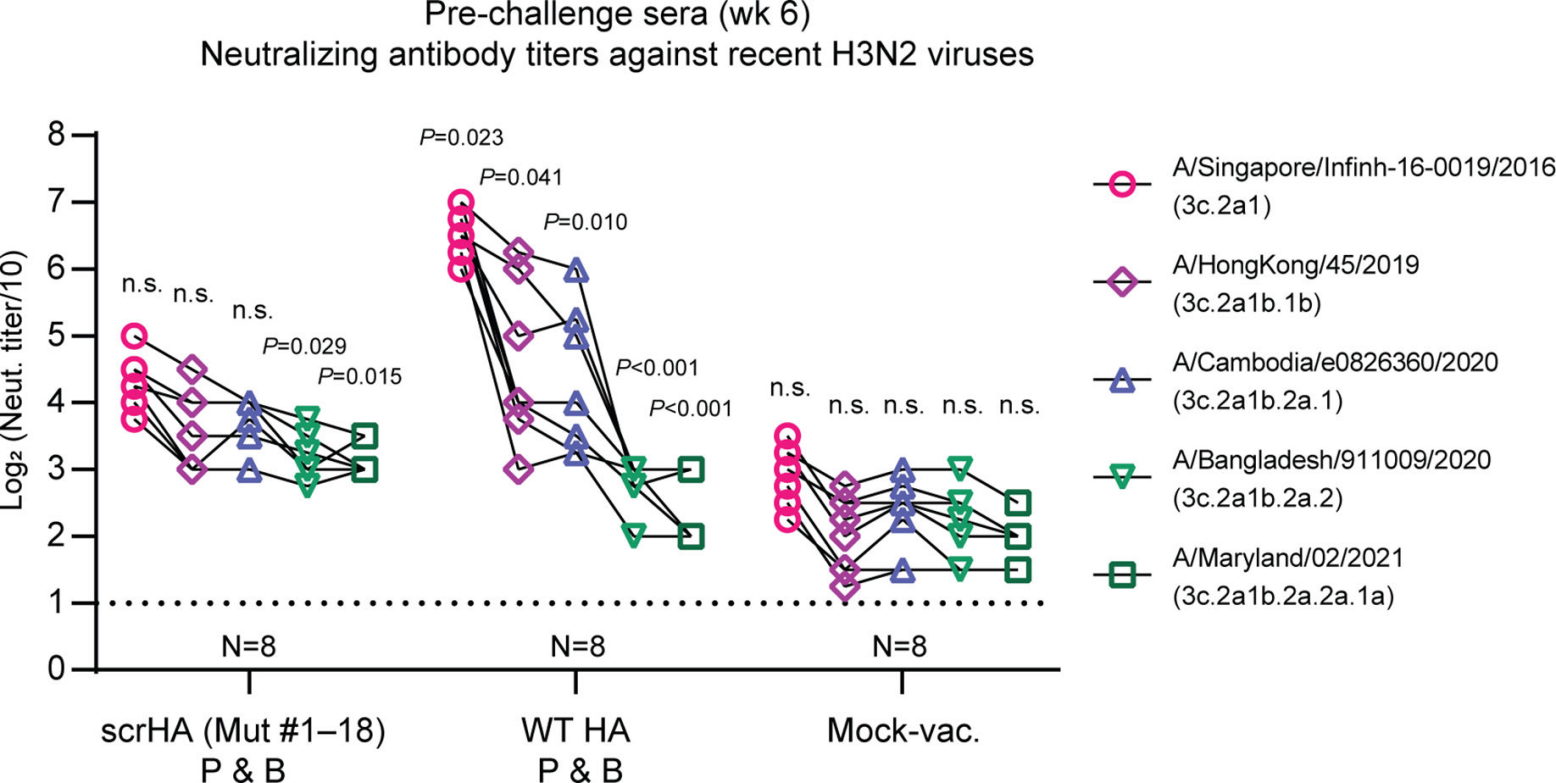
Characterization of scrHA-elicited neutralizing antibodies. Neutralizing antibody titers of pre-challenge sera from the scrHA (Mut #1–18) prime-and-boost (P & B) group (*N* = 8), the WT HA P & B group (*N* = 8), and the mock-vaccinated group (*N* = 8) were analyzed against recent H3N2 viruses from different antigenic clades. Data points show the values of individual animals connected by lines for each animal. Data shown are the geometric mean of duplicates. *P*-values are indicated compared to the titers against Tokyo/14 virus (shown in Fig. 2c) analyzed by a one-way analysis of variance and corrected for multi-group comparison by using Dunnett’s test. n.s., not significant. Dashed lines represent the detection limit of the assay.

To compare the cross-reactivity of the antibodies elicited by scrHA or WT HA vaccination, the pre-challenge sera from the prime-and-boosted group with scrHA (Mut #1–18) and WT HA were analyzed in a microneutralization assay with H3N2 viruses from recent antigenic clades (Fig. 4). The Tokyo/14 scrHA (Mut #1–18)-vaccinated group showed comparable levels of neutralizing titers against A/Singapore/Infinh-16–0019/2016 (Sing/16; clade 3 c.2a1), A/HongKong/45/2019 (HK/19; clade 3 c.2a1b.1b), and A/Cambodia/e0826360/2020 (Camb/2020; clade 3 c.2a1b.2a.1) viruses (Fig. 4, left) as against Tokyo/14 virus (Fig. 2c), although slightly reduced titers were detected against A/Bangladesh/911009/2020 virus (Bang/20; clade 3 c.2a1b.2a.2) and A/Maryland/02/2021 virus (MD/21; clade 3 c.2a1b.2a.2a.1a) (Fig. 4, left). In contrast, the Tokyo/14 WT HA-vaccinated group showed even higher neutralizing titers against Sing/16 virus than against Tokyo/14 virus (Fig. 4, middle; Fig. 2c), which could be explained by the fact that the Tokyo/14 and Sing/16 viruses belong to the most proximate antigenic clades and/or that higher background titers were detected against the Sing/16 virus in the mock-vaccinated group in this assay (Fig. 4, right). However, the neutralizing titers of the Tokyo/14 WT HA-vaccinated group declined steeply against the antigenically distinct HK/19, Camb/20, Bang/20, and MD/21 viruses (Fig. 4, middle). These results suggest that the neutralizing antibodies elicited by scrHA vaccination neutralize, albeit less potently, H3N2 viruses by targeting more the conserved region of H3HA, whereas those elicited by WT HA vaccination target clade-specific immunodominant epitopes.

### Antibodies targeting the immunosubdominant HA head domain and the stalk domain induced by scrHA may be involved in protective immunity

**FIG 5:**
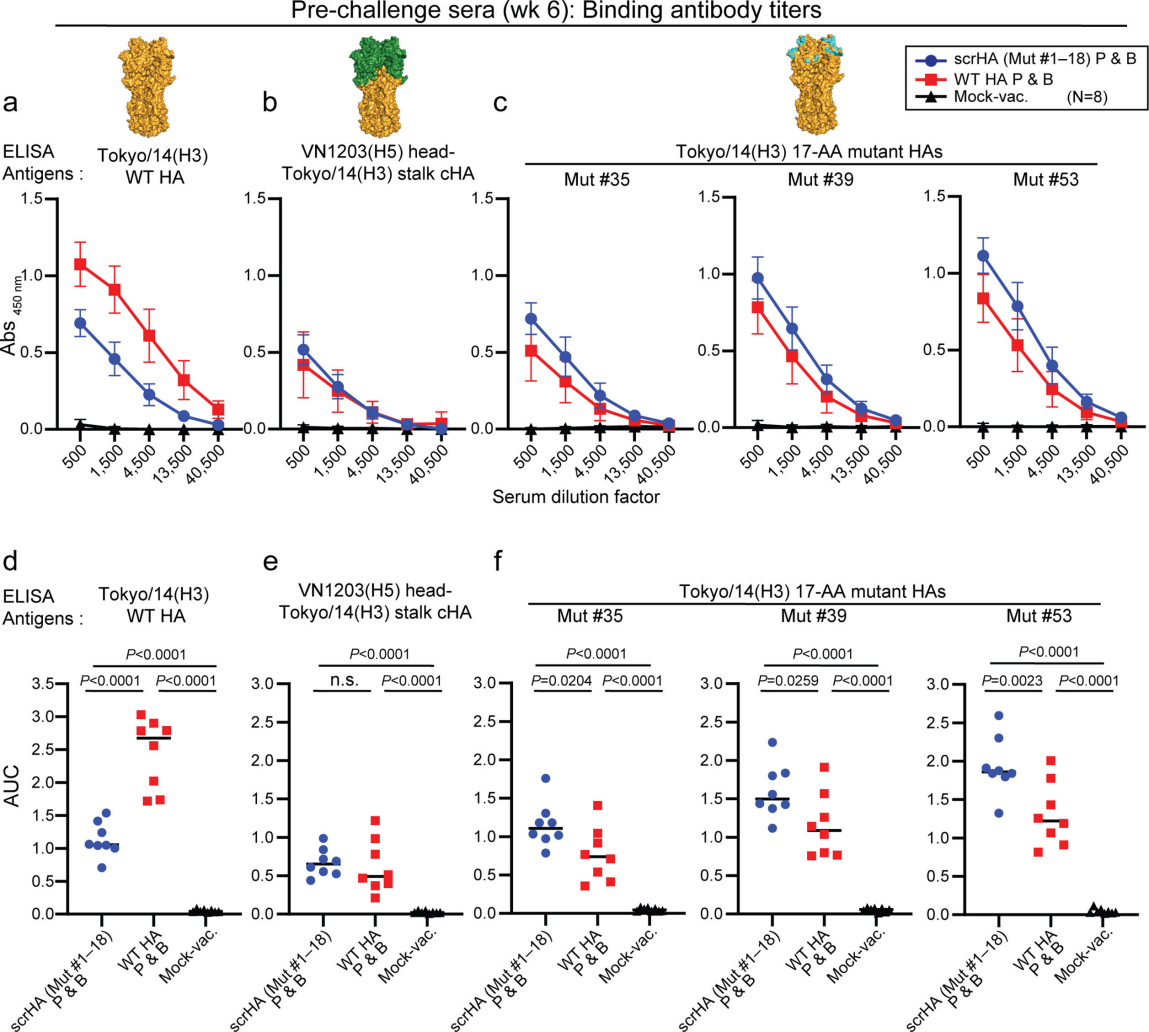
Characterization of scrHA-elicited HA-binding antibodies. (a–c) Binding antibody titers of pre-challenge sera from the scrHA (Mut #1–18) prime-and-boost (P & B) group (*N* = 8), the WT HA P & B group (*N* = 8), and the mock-vaccinated group (*N* = 8) were analyzed against Tokyo/14 wild-type HA (a), chimeric HA with the Vietnam/1203/2004 (VN1203; H5) head and Tokyo/14 stalk (b), and the Tokyo/14 17-AA mutant HAs (c; Mut #35, #39, and #53; the amino acid substitutions at 17 mutagenesis positions are shown in Supplementary Figure 6) in a cell-based ELISA by using full-length HA expressed on A549 cells. Data represent the means and SD of each group (*N* = 8). (d–f) AUCs (area under the curve) for individual animals (*N* = 8/group) in (a–c) were plotted. Bars show the median of the groups. Statistical analyses were performed by using a one-way analysis of variance and corrected for multi-group comparison by using Tukey’s test. n.s., not significant.

The pre-challenge serum neutralizing titers against Tokyo/14 virus were significantly lower in the group twice vaccinated with Tokyo/14 scrHA (Mut #1–18) than in the WT HA twice-vaccinated group (Fig. 2c). The nasal swab virus titers of the Tokyo/14 scrHA (Mut #1–18) group, however, were as effectively reduced as those observed in the WT HA-vaccinated group (Fig. 3a and c); this group also showed a significantly reduced body temperature spike on day 2 upon challenge with either homologous Tokyo/14 or heterologous Kansas/17 virus (Fig. 3b and d). We hypothesized that the antibodies elicited by scrHA vaccination were also involved in other protective mechanisms *in vivo*. Increasing evidence suggests that anti-HA stalk-binding antibodies effectively induce antibody-dependent cellular cytotoxicity (ADCC), mediated by Fc-Fc receptor engagement, which is competitively inhibited by hemagglutination-inhibiting (HAI) anti-HA head domain antibodies (i.e., antibodies targeting immunodominant head epitopes) and that the extent of ADCC is regulated by the relative proportion of ADCC-activating and ADCC-inhibiting antibodies (22 - 24). Also, HA stalk-specific antibodies, but not HA head-specific ones, induce reactive oxygen species production by neutrophils, resulting in optimal protection against influenza virus *in vivo* (25). To dissect the epitopes targeted by the HA-binding antibodies, we performed a cell-based enzyme-linked immunosorbent assay (ELISA) with WT Tokyo/14 HA, chimeric HA consisting of Tokyo/14 stalk and A/Vietnam/1203/2004 (VN1203; H5) HA head domain, and Tokyo/14 17-AA mutant HAs expressed on human lung epithelial cell line A549 cells. Binding IgG titers against WT full-length Tokyo/14 HA were significantly higher in the WT Tokyo/14 HA-vaccinated group than in the scrHA-vaccinated group (Fig. 5a and d). When the chimeric HA of the H5 head domain and the Tokyo/14 stalk domain was used to detect anti-stalk antibodies, however, binding IgG titers were detected at comparable levels for the two groups (Fig. 4b and e). In the same assay with the full-length VN1203(H5) HA, no binding antibody titers were detected in the vaccinated or mock-vaccinated groups (Supplementary Figure 5a and b). Notably, when the Tokyo/14 17-AA mutant HAs (#35, #39, and #53; Supplementary Figure 6), which are not included in the scrHA vaccine antigens, were used as ELISA antigens, higher binding titers were detected for the scrHA-vaccinated group than the WT HA-vaccinated group (Fig. 4c and f). Collectively, these results suggest that scrHA vaccination elicits higher binding antibody titers against immunosubdominant epitopes in the HA head domain compared to WT HA vaccination, whereas WT HA vaccination mainly targets immunodominant epitopes in the head and that both vaccines induce similar levels of anti-stalk antibodies.

## DISCUSSION

Universal influenza vaccines that target conserved HA epitopes have been sought to resolve vaccine mismatch issues. Here, based on the concept of “diluting out” immune responses targeting immunodominant strain-specific epitopes on the HA globular head and instead targeting conserved immunosubdominant HA epitopes, we developed HA antigens that are a mixture of mutants with divergent amino acid substitutions at immunodominant epitopes (scrHA) and examined their vaccine effects in a ferret model.

In the ferret challenge study with the homologous Tokyo/14 virus, the scrHA-vaccinated groups showed similarly reduced viral shedding to that of the WT HA-vaccinated group (Fig. 3a), although the pre-challenge serum neutralizing titers against this virus were lower than those of the WT HA-vaccinated group. Considering that the immunodominant epitopes in the HA head are drastically altered in scrHA antigens, it is remarkable that the scrHA and WT HA vaccines similarly reduced homologous virus replication in nasal mucosa, suggesting that different mechanisms may be involved in the protective immune responses induced by scrHA and WT HA vaccination. Upon heterologous Kansas/17 virus challenge, scrHA vaccination did not show an advantage over WT HA immunization in terms of reducing nasal swab virus titers despite our conceptual expectation but inhibited virus replication to similar levels as Tokyo/14 WT HA vaccination. Notably, however, one of the scrHA-vaccinated groups (Mut #1–18, prime-and-boost regimen) showed a significantly lower increase in body temperature upon homologous or heterologous virus challenge, suggesting that scrHA vaccination may mount immune responses to reduce disease severity. Our finding that the scrHA containing 18 mutants, but not the mixture of 9 mutants, reduced the increase in body temperature (Fig. 3b and d) suggests that some degree of amino acid variety is needed at the immunodominant locations for scrHA antigens to induce protective immune responses to reduce disease severity, although the mechanism is unknown. Future experiments are warranted to determine the minimum variety needed to induce such protective immune responses so that the number of mutants required can be reduced or the most immunodominant epitopes to be substituted can be narrowed down, which would result in reduced vaccine preparation workloads.

The ferrets vaccinated with scrHA did not elicit high neutralizing titers against the homologous Tokyo/14 virus, compared with the WT HA-vaccinated group (Fig. 2c). This could be due to the fact that all the mutant HAs that comprised the scrHA antigens were isolated as antigenically distinct mutants from WT HA in the neutralization assay with Tokyo/14 ferret antisera (Fig. 1d). Also, notably, several animal model studies have demonstrated that anti-stalk antibodies have only low neutralizing titers *in vitro* compared with anti-head antibodies, despite equivalent protective potency *in vivo* (22, 26 - 28). The “moderate” neutralizing titers against homologous Tokyo/14 induced by scrHA vaccination were, however, also similar against heterologous Kansas/17 virus and antigenically distinct recent H3N2 viruses, contrasting with the dramatic titer decline against those viruses observed for the WT HA-vaccinated group (Fig. 2c and 4). These results suggest that the scrHA vaccine elicits neutralizing antibodies that target more conserved epitopes, whereas WT HA vaccination induces neutralizing antibodies targeting strain-specific epitopes.

The protective immunity observed *in vivo* in the scrHA-vaccinated group despite lower *in vitro* neutralizing antibody titers compared to the WT HA-vaccinated group suggests some mechanisms other than neutralizing antibodies are involved in the scrHA-induced immune responses. We observed that scrHA immunization elicited reduced amounts of binding antibodies against the immunodominant HA head domain and increased amounts of antibodies against the immunosubdominant head epitopes compared with WT HA-immunization, whereas binding antibodies against the immunosubdominant HA stalk were induced at comparable levels for the two groups (Fig. 5). These results demonstrate that the scrHA vaccine shifted the antibody responses from the immunodominant head epitopes to immunosubdominant head epitopes.

There are a couple of limitations in this study. First, to assess vaccine effectiveness, humoral immunity was mainly analyzed as neutralizing antibody titers, whereas T-cell-mediated immune responses or Fc-dependent effector functions were not directly analyzed due to the lack of ferret-specific reagents. Second, Alhydrogel, a typical Th2-biased adjuvant, was used to induce neutralizing antibodies; however, vaccine formulation with different adjuvants could result in different outcomes. Finally, we did not examine how the scrHA-elicited antibodies might be boosted to keep targeting the immunosubdominant epitopes (i.e., whether the same scrHA antigen could be used or another set of mutants would need to be formulated as a “booster” shot) after the antibody titer has waned over time.

In summary, although the scrHA vaccine strategy was only comparable to the WT HA vaccine in terms of reducing heterologous virus shedding in the upper respiratory mucosa, it reduced body temperature increase upon virus challenge, unlike the WT HA vaccine. Therefore, the goal of “diluting out” immunodominant epitopes on the HA head domain could be achieved with scrHA through redirection toward immunosubdominant head epitopes, which may contribute to inducing more optimal Fc-dependent effector functions *in vivo*.

## MATERIALS AND METHODS

### Cells

Madin-Darby canine kidney (MDCK) cells were maintained in Eagle’s minimal essential medium (MEM) containing 5% newborn calf serum (NCS) and antibiotics. hCK cells (29) were maintained in Eagle’s MEM containing 5% NCS in the presence of 2 µg/mL puromycin (Invivogen, San Diego, CA, USA) and 10 µg/mL blasticidin (InvivoGen). Human embryonic kidney 293T cells were cultured in Dulbecco’s modified Eagle’s medium (DMEM) containing 10% fetal bovine serum (FBS). SIAT1-MDCK cells were maintained in the presence of 1 mg/mL geneticin (G418; InvivoGen) in DMEM containing 5% FBS and antibiotics. A549 cells were maintained in Dulbecco’s modified Eagle’s medium: nutrient mixture F-12 (DMEM/F-12) containing 10% FBS. These cell lines were incubated at 37°C with 5% CO_2_. Expi293F cells (Thermo Fisher Scientific, Waltham, MA, USA) were maintained in Expi293 expression media (Thermo Fisher Scientific) in spinner flasks, at a spinner speed of 120 rpm, at 37°C under 8% CO_2_. All cell lines were regularly tested for mycoplasma contamination by using PCR and were confirmed to be mycoplasma free.

### Viruses

H3N2 influenza virus A/Netherlands/399/1993 (WU95 cluster) was kindly provided by Dr. Ron A.M. Fouchier at Erasmus Medical Centre, Rotterdam, The Netherlands. A/Tokyo/UT-IMS2-1/2014_PR8HY virus and its 17-AA HA mutant viruses (Mut #1–18) cloned from the library virus, A/Kansas/14/2017_PR8HY, A/Aichi/2/1968_PR8HY, A/Singapore/Infinh-16-0019/2016_PR8HY, A/Hong Kong/45/2019_PR8HY, A/Cambodia/e0826360/2020_PR8HY, A/Bangladesh/911009/2020_PR8HY, and A/Maryland/02/2021_PR8HY viruses were generated by reverse genetics (30) with the HA and NA segments from the homologous virus and the other segments from the PR8 high-yield backbone (21) and were propagated in hCK cells in MEM containing 0.5 µg/mL *N-p*-tosyl-L-phenylalanine chloromethyl ketone (TPCK)-treated trypsin. None of the viruses used in this study was isolated or amplified in chicken embryonic eggs.

### Recombinant protein expression and purification

Soluble-form recombinant HAs (rHA) consisting of the HA signal peptide and ectodomain (amino acid residues 1–505; H3 numbering) with stabilizing mutations to form disulfide bonds (T30C and Q376C), followed by a T4 foldon trimerization domain and a hexa-histidine tag at the C-terminus (31), were cloned into pCAGGS plasmids. Proteins were expressed in Expi293F cells (Thermo Fisher Scientific) and purified by using TALON metal affinity resin (TaKaRa Bio USA, Ann Arbor, MI, USA).

### Animal experiments

All experiments with ferrets were performed in accordance with the guidelines set by the Institutional Animal Care and Use Committee at the University of Wisconsin-Madison. The protocol was approved by the Animal Care and Use Committee of the University of Wisconsin-Madison (protocol number V6426).

### Immunization and virus challenge of ferrets

Ferrets (4–6-mo-old females; TripleF) were confirmed to be seronegative in a hemagglutination inhibition (pH1N1 or flu B viruses) or micro-neutralization assay (H3N2 viruses) with recent circulating influenza viruses before immunization. Animals were intramuscularly immunized with 15 µg of purified rHA protein (diluted in 100 µL of PBS) and adjuvanted with Alhydrogel (2% solution, 100 µL; InvivoGen). Animals were serially bled via the jugular vein under ketamine and dexdomitor anesthesia. Then, the animals were intranasally inoculated with 10^6^ pfu of A/Tokyo/UT-IMS2-1/2014_PR8HY virus or A/Kansas/14/2017_PR8HY virus under anesthesia. Nasal swabs were taken daily, alternately from the left and right nostrils for 7 days. During this period, body temperature was measured by using an implantable subcutaneous temperature transponder (Bio Medic Data Systems, Seaford, DE, USA). Nasal swabs were resuspended in 1 mL of MEM containing antibiotics and kept at –80°C until titration by means of plaque assays in hCK cells.

### Micro-neutralization assay

Virus-neutralizing antibody titers against H3N2 influenza viruses were evaluated in serum samples. Serum samples were treated with receptor-destroying enzyme (Denka seiken, Tokyo, Japan) at 37°C for 20 h, inactivated at 56°C for 1 h, and diluted 1:10 in PBS. Twofold serial dilutions of sera were prepared in MEM, and each dilution was incubated with the same volume of virus diluent (100 TCID_50_/50 µL) in MEM containing 1 µg/mL of TPCK-trypsin at room temperature for 1 h. The serum/virus mixture was added to 100% confluent SIAT1-MDCK cells that were plated a day prior in 96-well plates. The cells were incubated for 5 days at 33°C and then cytopathic effect (CPE) was microscopically assessed by eye. Virus neutralization titers were determined as the reciprocal of the highest serum dilution that completely prevented CPE. Each sample was analyzed in duplicate for geometric mean titers.

### Full-length or chimeric HA expression plasmid construction

A/Tokyo/UT-IMS2-1/2014 WT HA (H3HA) as well as 17-AA mutant HAs, and detoxified A/Vietnam/1203/2004 (H5HA; the cleavage site RERRRKKR/G was substituted with RETR/G, the slash indicates the cleavage site) (32) were cloned into pCAGGS plasmid. cHA with the N-terminal signal peptide, stalk domain, transmembrane domain, and intracellular domain (aa 1–52 and aa 277–550, H3 numbering) from A/Tokyo/UT-IMS2-1/2014 HA (H3 HA) and the head domain (aa 52–277; Cys52 and Cys277 are conserved cysteine residues among group 1 and group 2 HAs that form a disulfide bond) (2) from detoxified A/Vietnam/1203/2004 (H5 HA) was cloned into pCAGGS plasmid.

### Cell-based ELISA

The cell-based ELISA was performed using A549 cells transiently expressing HA by pCAGGS plasmid transfection. A549 cells were seeded in poly-_L_-lysine-coated 96-well plates at a density of 1 × 10^4^ cells/well. At 24 h post-seeding, the cells were transfected with 100 ng/well of pCAGGS plasmid-encoding influenza HA or cHA and 0.2 µL of TransIT-X2 (Mirus Bio, Madison, WI, USA). At 48 h post-transfection, the cells were fixed with 50 µL/well of phosphate-buffered saline (PBS) containing 4% paraformaldehyde at room temperature for 15 min. After the plates were blocked with PBS containing 1% BSA at 4°C overnight, they were incubated with heat-inactivated (56°C for 30 min) ferret serum that was threefold serially diluted in PBS containing 0.5% BSA and 0.05% Tween 20 (PBS-BT). After a 1-h incubation at room temperature, the plates were washed with PBS containing 0.1% Tween 20 (PBS-T) four times and then incubated with antiferret IgG (H + L) secondary antibody conjugated with horseradish peroxidase (Novus, Saint Charles, MO, USA; 1:10,000 dilution in PBS-BT) at room temperature for 1 h. Then, the plates were washed four times with PBS-T and developed with 1-Step Ultra TMB-ELISA Substrate Solution (Thermo Scientific). After a 15-min incubation, the reaction was stopped with the addition of 1 N sulfuric acid. The absorbance was measured immediately at a wavelength of 450 nm.

## SUPPLEMENTAL MATERIAL

Supplemental information mbio.00622-23-s0001.docx Supplementary notes and figures.

ASM does not own the copyrights to Supplemental Material that may be linked to, or accessed through, an article. The authors have granted ASM a non-exclusive, world-wide license to publish the Supplemental Material files. Please contact the corresponding author directly for reuse.
