# Supplementary material for: Influenza H3 hemagglutinin vaccine with scrambled immunodominant epitopes elicits antibodies directed toward immunosubdominant head epitopes
Source: mBio. 2023 Jul 19;14(4):e00622-23. doi: 10.1128/mbio.00622-23 (PMC10470489; doi:10.1128/mbio.00622-23)
Supplement: Supplemental information — Supplementary notes and figures. [file mbio.00622-23-s0001.docx]

**Supplementary information of “Influenza H3 hemagglutinin vaccine with scrambled immunodominant epitopes elicits antibodies directed toward immunosubdominant head epitopes”**

Shiho Chiba^1^, Huihui Kong^1^, Gabriele Neumann^1^, and Yoshihiro Kawaoka^1,2,3,4^

^1^Influenza Research Institute, Department of Pathobiological Sciences, School of Veterinary Medicine, University of Wisconsin-Madison, Madison, Wisconsin, USA

^2^Division of Virology, Department of Microbiology and Immunology, Institute of Medical Science, University of Tokyo, Tokyo, Japan

^3^The Research Center for Global Viral Diseases, National Center for Global Health and Medicine Research Institute, Tokyo, Japan.

^4^Pandemic Preparedness, Infection and Advanced Research Center (UTOPIA), The University of Tokyo, Tokyo, Japan

Contents:

Supplementary Notes: pp. 2–5

Supplementary Figures: pp. 6–15

References: p. 16

**Supplementary note 1. Selection of antigenically divergent candidate mutant HAs for scrHA vaccine antigens from the Tokyo/14 17-AA library virus**

To isolate antigenically distinct mutant HAs from the wild-type (WT) Tokyo/14 HA by using 17-AA mutant HA library viruses, two different strategies were employed:

(1) The Tokyo/14 17-AA mutant HA library virus was screened in a plaque assay supplemented with 12 human sera mixed in the same ratio collected in 2015–2016 that had been found to be reactive with Tokyo/14 WT virus in a focus reduction assay (the sera are listed in Supplementary Table 1; purchased from Lampire Biological Laboratories); the individual mutant viruses were isolated by plaque picking and grown in hCK cells^1^, and viral RNA was extracted from the culture supernatant and sequenced. By utilizing 5 mL of mutant HA library virus (10^4^ pfu/mL), 63 clones of genetically/antigenically divergent HA mutant viruses were isolated as replication-competent mutant viruses (approx. 800-fold plaque number reduction with serum pressure compared to without serum). Mutants #1 to #3 (used for scrHA) and mutants #35, #39, and #53 (used in the cell-based ELISA) were isolated through this process.

**Supplementary Table 1. Human sera used to screen the Tokyo/14 17-AA HA library virus**

| Serum Lot# | Date of collection | Age of the Donor | Year of Birth of the Donor | Serum Titer against A/Tokyo/UT-IMS2-1/2014 (FRA)* |
| --- | --- | --- | --- | --- |
| LQ236255 | 4/8/2015 | 28 | 1987 | 80 |
| LQ236231 | 4/8/2015 | 29 | 1986 | 160 |
| LQ236244 | 4/8/2015 | 31 | 1984 | 40 |
| LQ236263 | 4/8/2015 | 32 | 1983 | 320 |
| LQ236220 | 4/8/2015 | 52 | 1963 | 160 |
| LQ236266 | 4/8/2015 | 54 | 1961 | 160 |
| LQ236226 | 4/8/2015 | 58 | 1957 | 80 |
| LS 88 26189A | 7/25/2016 | 19 | 1997 | >512 |
| LS 55 53405A | 7/27/2016 | 22 | 1994 | >512 |
| LS 23 85911A | 7/27/2016 | 30 | 1986 | >512 |
| LS 88 26222A | 7/26/2016 | 35 | 1981 | >512 |
| LS 88 26185A | 7/25/2016 | 39 | 1977 | >512 |

*** FRA, focus reduction assay.**

(2) Not all the mutant HAs isolated through the strategy described above were expressed as soluble form recombinant HA (rHA) proteins in the human-derived Expi293F cell line at levels feasible to be used as animal vaccination antigens, despite replication-competency in cells as mutant virus clones. Therefore, we employed another strategy to identify stable mutant HAs as rHA proteins. To enrich for functionally/structurally stable HAs, aliquots of the Tokyo/14 17-AA HA virus library were serially passaged five times in individual wells in hCK cells plated in 96-well plates at 37 °C. After five consecutive passages, viral RNA was extracted from the culture supernatant, and the most dominant population of HA segment was cloned from individual wells. Beginning with approximately 1,000 individual passage lines, 242 genetically unique clones were isolated after mutants with identical sequences were eliminated. Then, the isolated mutant HAs were individually cloned into pCAGGS plasmid as rHA to test expression levels in Expi293F cells; 30 HA mutants with relatively high expression levels were selected for further study. These mutants were also cloned into pCAGGS plasmid as full-length HAs for antigenic analysis. Reactivity against human H3N2 convalescent plasma collected in 2016–2018 was examined in a cell-based ELISA and normalized to reactivity with an anti-HA stem monoclonal antibody (mAb; 042 100809 2F04 ^2^). Mutants with high reactivity against at least 4 of 5 convalescent sera (i.e., those similar to WT HA) were eliminated from the candidates. Finally, mutants whose rHAs were unstable during purification were eliminated. Mutants #4 to #18 were isolated through this process.

**Supplementary Table 2. The reactivity of candidate Tokyo/14 17-AA HA mutants against human convalescent plasma**

**
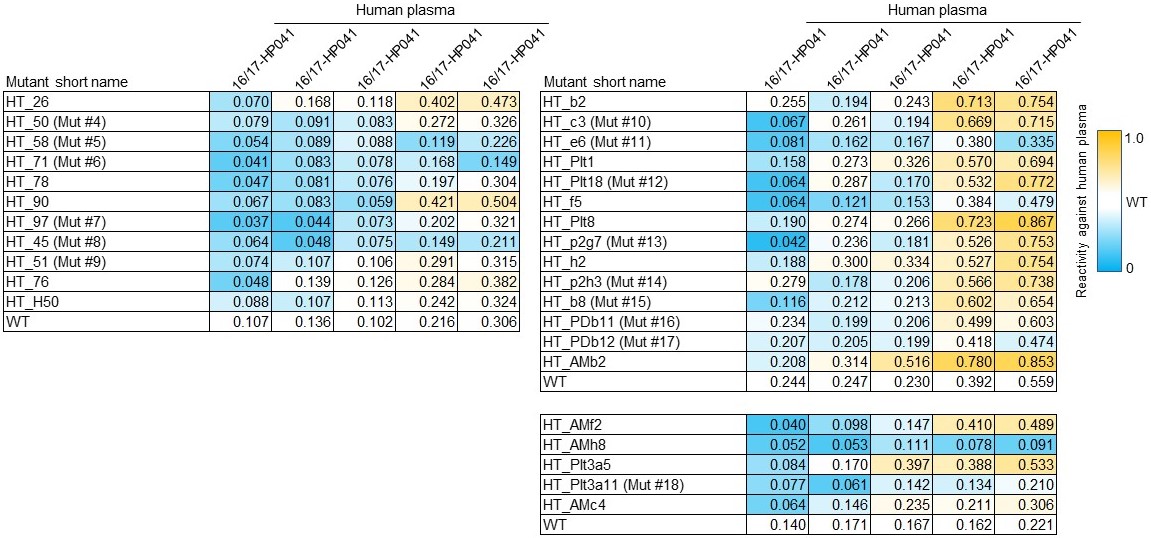
**

The signal intensity detected in the cell-based ELISA with each human convalescent plasma sample was normalized to that detected with the anti-HA stalk monoclonal antibody. The values were compared with wild-type (WT) HA within each experimental batch. Higher or lower reactivity against each plasma compared to WT HA is colored orange or light blue, respectively.


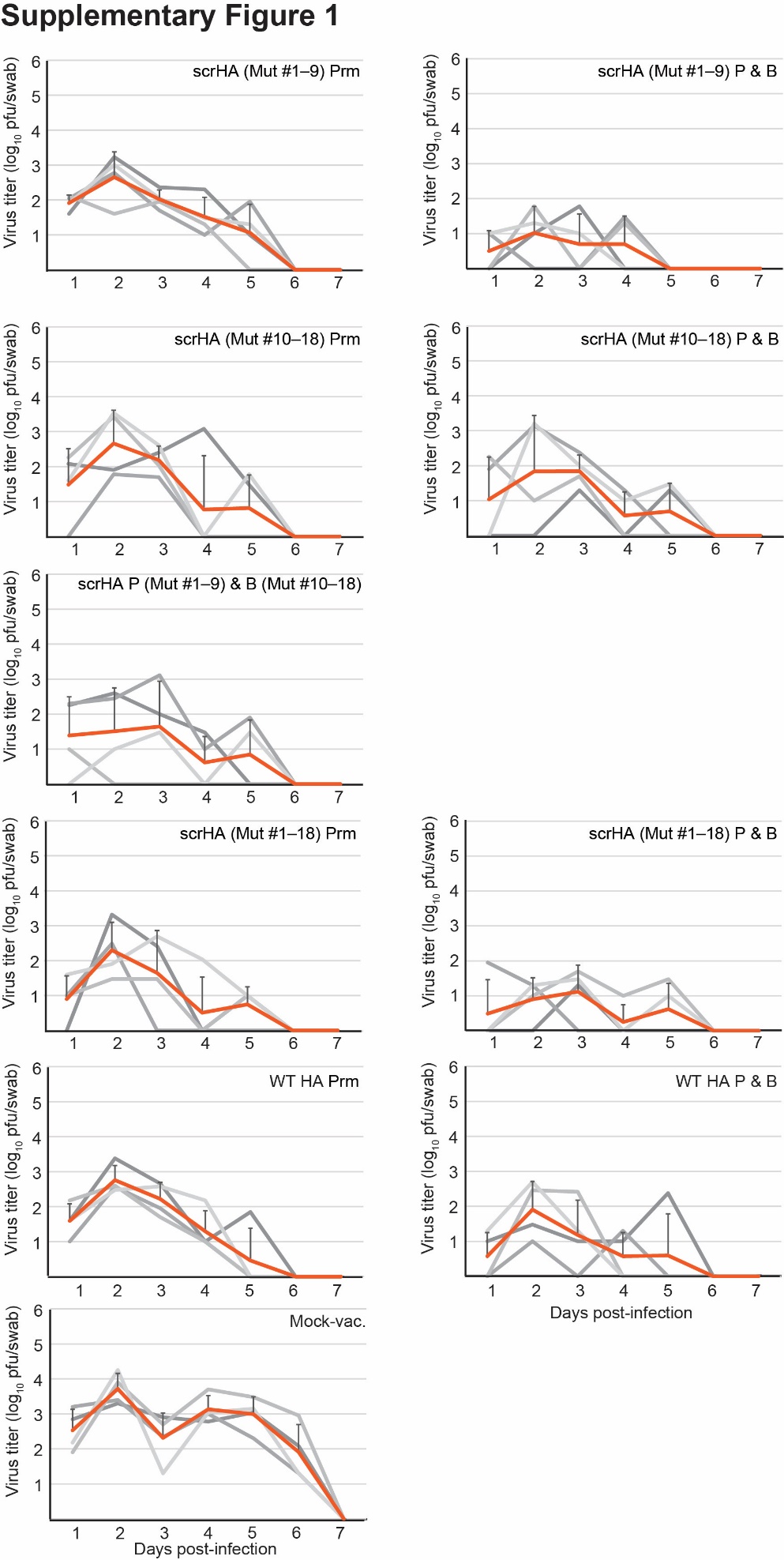


**Supplementary Figure 1. Virus replication upon Tokyo/14 virus challenge.** Vaccinated or mock-vaccinated ferrets (N=4/group) were intranasally infected with 10^6^ pfu of A/Tokyo/UT-IMS2-1/2014 virus. Nasal swab titer data of individual animals (gray lines) and the average (red line) and SD of the group are shown in each panel. Prm, prime-only regimen; P & B, prime-and-boost regimen.


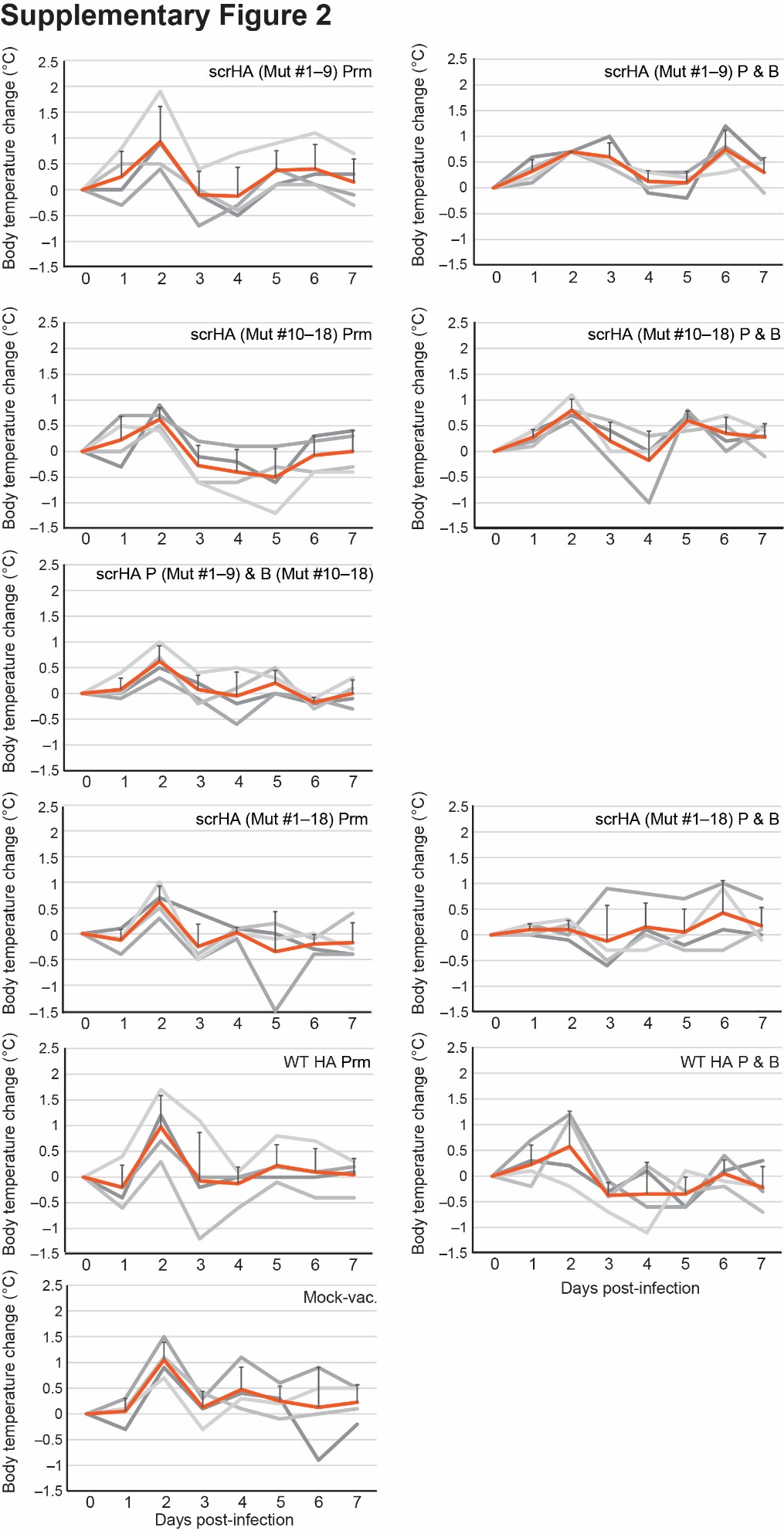


**Supplementary Figure 2. Body temperature change upon Tokyo/14 virus challenge.** Vaccinated or mock-vaccinated ferrets (N=4/group) were intranasally infected with 10^6^ pfu of A/Tokyo/UT-IMS2-1/2014 virus. Body temperature data of individual animals (gray lines) and the average (red line) and SD of the group are shown in each panel. Prm, prime-only regimen; P & B, prime-and-boost regimen.


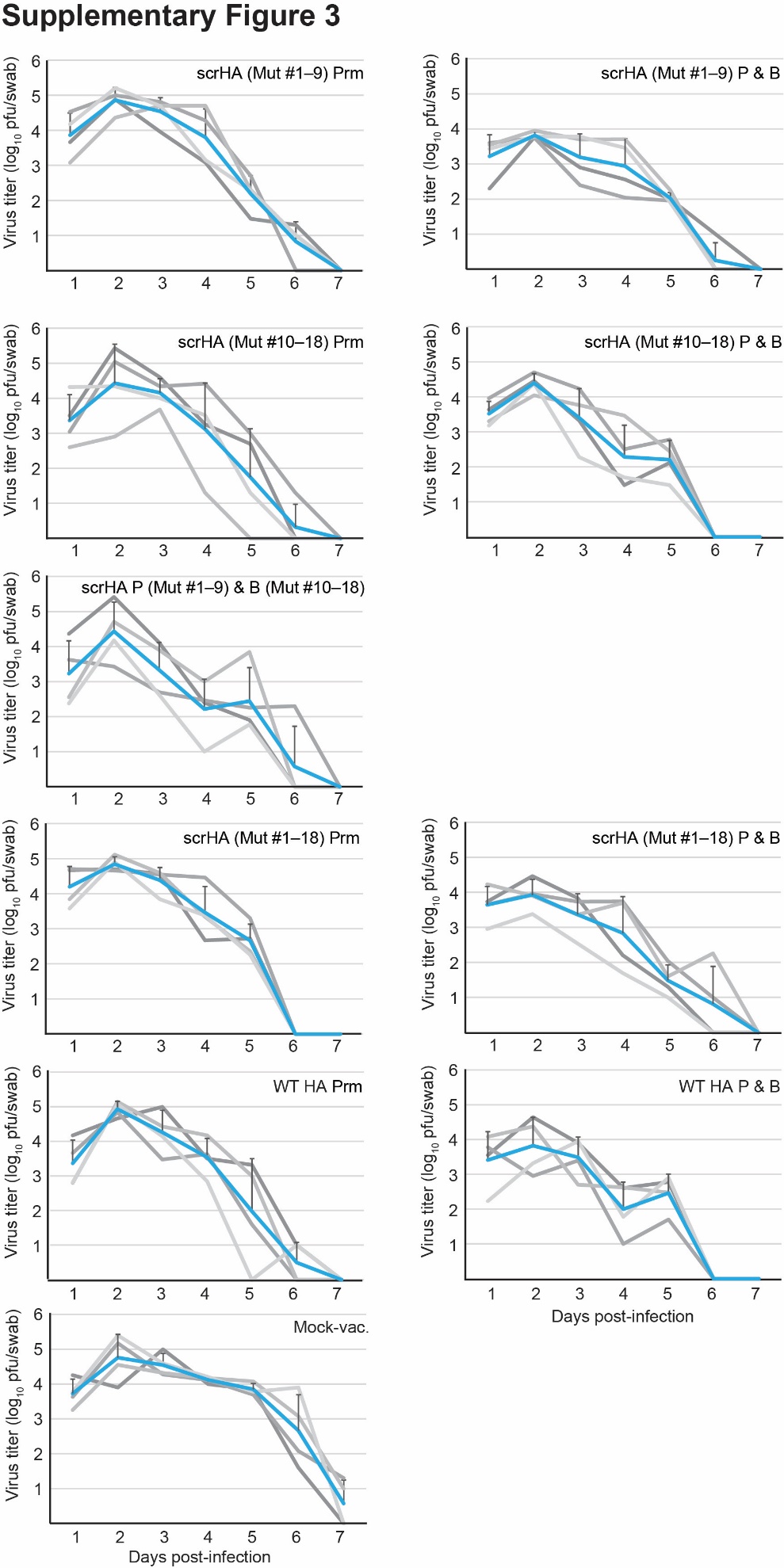


**Supplementary Figure 3. Virus replication upon Kansas/17 virus challenge.** Vaccinated or mock-vaccinated ferrets (N=4/group) were intranasally infected with 10^6^ pfu of A/Kansas/14/2017 virus. Nasal swab titer data of individual animals (gray lines) and the average (blue line) and SD of the group are shown in each panel. Prm, prime-only regimen; P & B, prime-and-boost regimen.


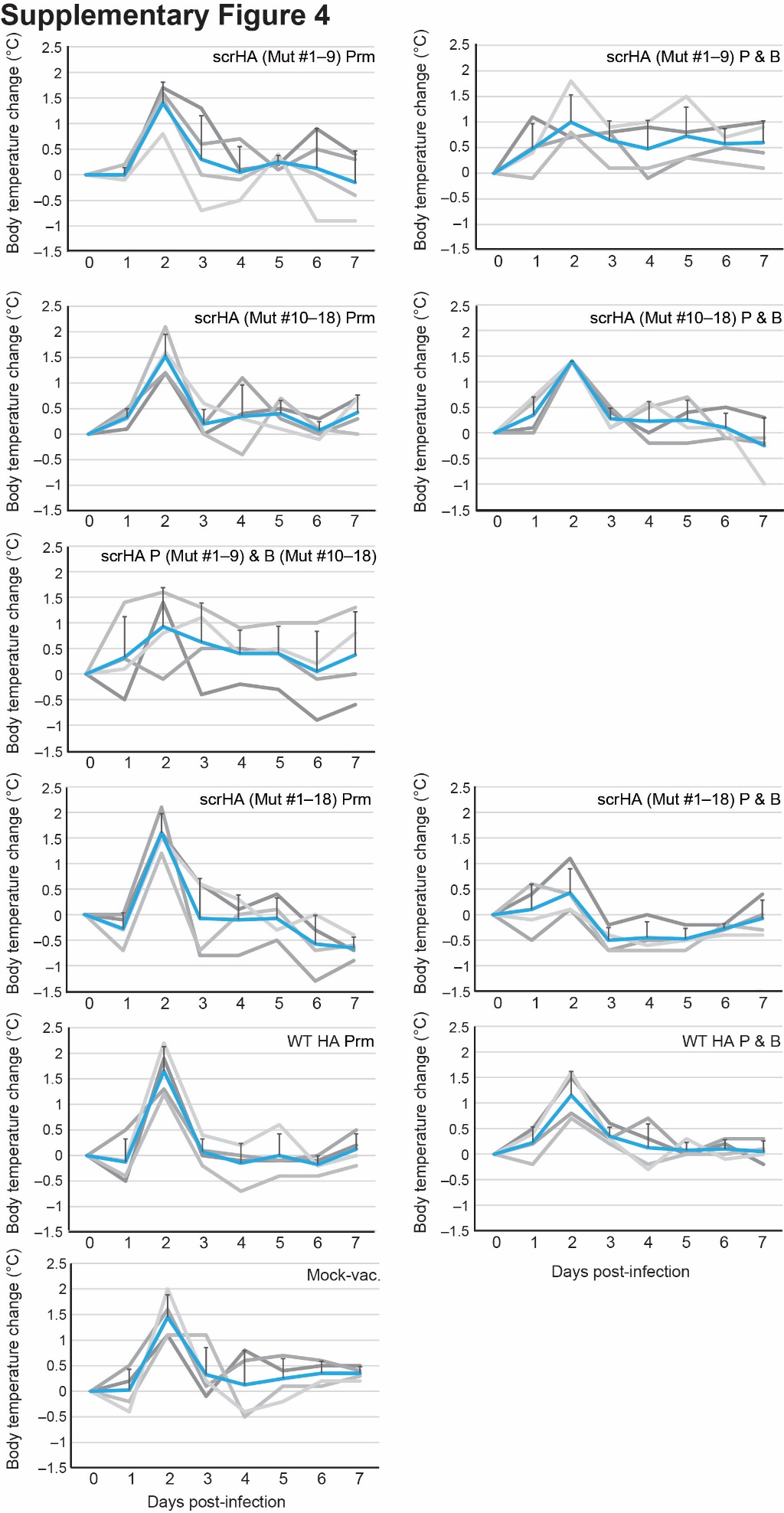


**Supplementary Figure 4. Body temperature change upon Kansas/17 virus challenge.** Vaccinated or mock-vaccinated ferrets (N=4/group) were intranasally infected with 10^6^ pfu of A/Kansas/14/2017 virus. Body temperature data of individual animals (gray lines) and the average (blue line) and SD of the group are shown in each panel. Prm, prime-only regimen; P & B, prime-and-boost regimen.


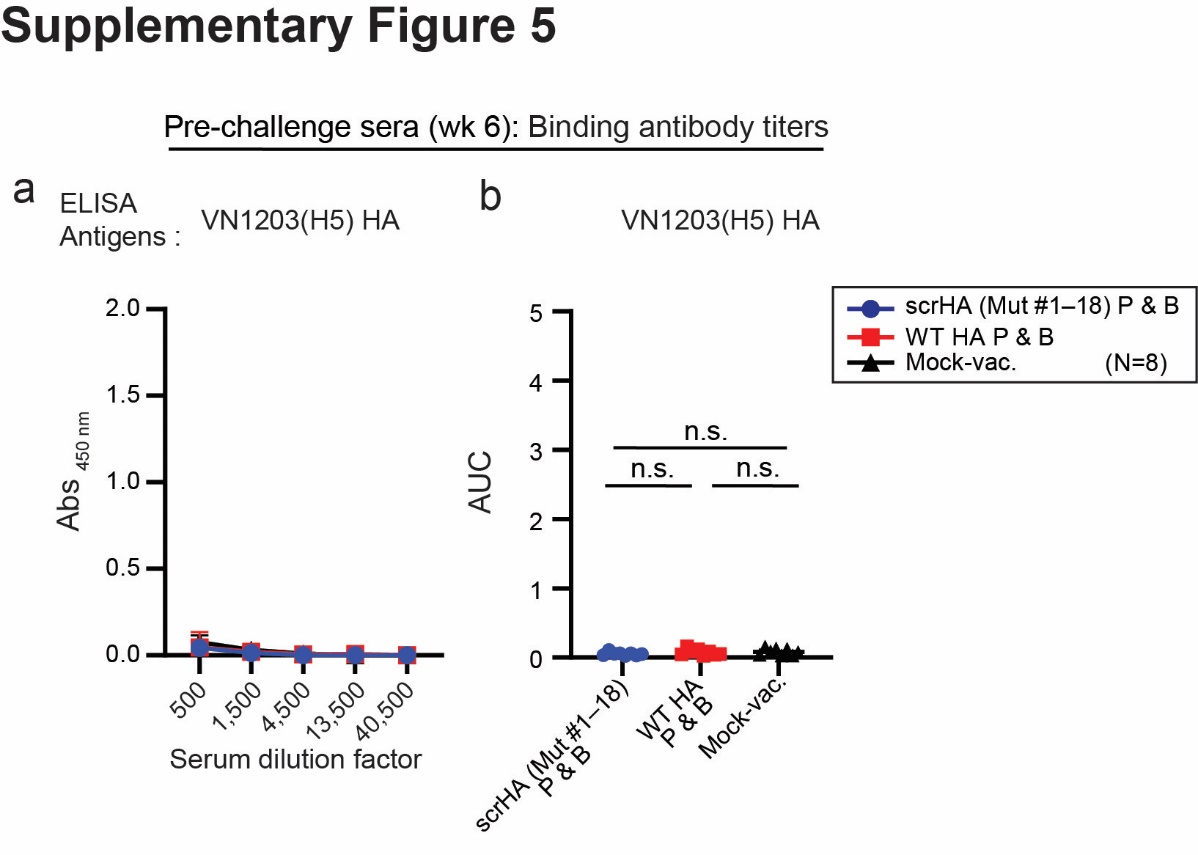


**Supplementary Figure 5. Characterization of scrHA-elicited HA-binding antibodies.** (a) Binding antibody titers of pre-challenge sera from the scrHA (Mut #1–18) prime and boost (P & B) group (N=8), the WT HA P & B group (N=8), and the mock-vaccinated group (N=8) were analyzed against Vietnam/1203/2004 (VN1203; H5) HA in a cell-based ELISA by using full-length HA expressed on A549 cells. Data represent the means and SD of each group (N=8). (b) AUCs (area under the curve) for individual animals (N=8/group) in (a) were plotted. Bars show the median of the groups. Statistical analyses were performed by using a one-way analysis of variance (ANOVA) and corrected for multi-group comparison by using Tukey’s test. n.s., not significant.

**
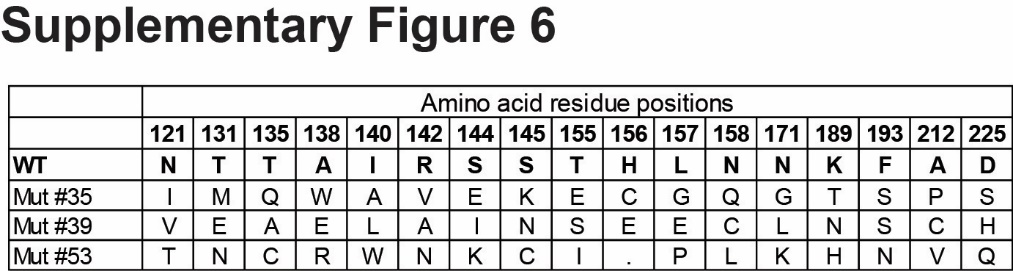
**

**Supplementary Figure 6. Tokyo/14 17-AA mutant HAs used in the cell-based ELISA.** The table shows the amino acid residues at the 17 mutagenesis positions in wild-type A/Tokyo/UT-IMS2-1/2014 (Tokyo/14) HA and the amino acid substitutions in mutant HAs #35, #39, and #53. The dot indicates the identical amino acid residue to that found in wild-type HA.

**References**

1 Takada, K. *et al.* A humanized MDCK cell line for the efficient isolation and propagation of human influenza viruses. *Nat Microbiol* **4**, 1268-1273, doi:10.1038/s41564-019-0433-6 (2019).

2 Henry Dunand, C. J. *et al.* Preexisting human antibodies neutralize recently emerged H7N9 influenza strains. *J Clin Invest* **125**, 1255-1268, doi:10.1172/JCI74374 (2015).
